# The Association Between Vitamin B12 Deficiency and Diabetic Foot Ulcer in Type 2 Diabetic Patients in Qassim Province, Saudi Arabia: A Case-Control Study

**DOI:** 10.7759/cureus.68598

**Published:** 2024-09-04

**Authors:** Dalal M Alruqayi, Jolan S Alsaud, Jood M Alsogaihi, Wateen Alsawyan, Latifah Y Almutlaq, Aishah Alsuhaibani, Ahmed Alshammari, Habeebah Alghadouni, Mariam Alharbi

**Affiliations:** 1 Department of Medicine, Qassim Health Cluster, Qassim, SAU; 2 Department of Family Medicine, Qassim Health Cluster, Qassim, SAU; 3 College of Medicine, Qassim University, Qassim, SAU; 4 Department of Surgery, Qassim Health Cluster, Qassim, SAU; 5 Department of Endocrinology, Qassim Health Cluster, Qassim, SAU; 6 Department of Internal Medicine, College of Medicine, Qassim University, Qassim, SAU

**Keywords:** vitamin b12 deficiency, type 2 diabetes mellitus, retrospective case-control study, neuropathy and ulcer development, diabetic foot ulcer

## Abstract

Background

Diabetic foot ulcer (DFU) is a major complication of diabetes with many identified risk factors. These include poor control of diabetes, cardiovascular disease, smoking, and end-stage kidney disease. This study aims to shed light on the micronutrient status of diabetic patients and its effect on DFU, particularly, the association between vitamin B12 deficiency and DFU.

Methodology

This retrospective case-control study included adults in Buraydah who were at least 18 years old and had type 2 diabetes mellitus. Data were obtained from the electronic files of the patients who visited the diabetes center from January 2018 to August 2023 and were analyzed using SPSS version 27.0.1 (IBM Corp., Armonk, NY, USA).

Results

The research involved 221 participants, with 114 controls (individuals with diabetes but no DFU), and 107 cases (individuals with diabetes affected by DFU). Vitamin B12 levels varied, with 79.2% falling within the normal range of 187-883 pg/mL. The average age of cases (58.5 years, SD = 11.3) was notably higher than that of controls (54.1 years, SD = 14.1). Glycated hemoglobin levels were significantly higher in cases (8.7, SD = 2.0) compared to controls (7.6, SD = 2.2) (p < 0.001). Regarding physical activity, cases showed a significantly higher percentage of inactivity (62.1%) compared to controls (39.1%) (p = 0.046). Neuropathy exhibited a significant association with ulcer development, with 59.1% of cases having neuropathy compared to 23.5% of controls (p < 0.001). Furthermore, complications such as dry foot and fissures (60.0% vs. 6.3%), Charcot joint (36.8% vs. 12.2%), and foot trauma (40.9% vs. 3.9%) were significantly more prevalent in cases compared to controls (p < 0.001 for all).

Conclusions

The significant associations observed with advanced age, uncontrolled diabetes, longer diabetes duration, neuropathy, and specific foot complications underscore the multifactorial nature of ulcer development. The normal levels of vitamin B12 in most patients reflect no positive impact of normalized vitamin B12 levels on DFU. However, further observational studies with multiple vitamin B12 readings over a longer period are needed to establish its association with DFU development.

## Introduction

Diabetic foot ulcer (DFU) is a major cause of morbidity and mortality in diabetic patients, with approximately 18.6 million people affected by DFU worldwide [1]. DFU is defined as a break in the epidermis and at least part of the dermis layer of the skin of a diabetic patient. It is classified into neurologic, ischemic, or mixed neuroischemic based on underlying conditions such as peripheral neuropathy or peripheral artery disease. Repetitive trauma resulting from increased pressure on the weight-bearing surface of the foot, friction from ill-fitting footwear, or unnoticed injuries due to sensory neuropathy mainly cause DFU. The most important risk factors for DFU are chronically elevated glycated hemoglobin (HbA1c), smoking, cardiovascular disease, end-stage kidney disease, and chronic kidney disease [2]. The main lines of treatment for DFU are surgical debridement, reducing pressure at the weight-bearing site, and treatment of any associated ischemia or infection [1]. Preventive strategies include identifying patients with at-risk feet and regular inspection and examination of those patients, providing structural education for the patients and their families, and treating risk factors for ulceration [3].

Diabetes mellitus (DM) is a chronic disease with many nutritional consequences. Patients with DM have disturbed levels of multiple vitamins and minerals. The nutritional status of patients with DM may contribute to their risk of developing diabetes complications [4,5]. Studies have shown a possible association between low vitamin D levels and DFU and diabetic foot infections [6]. A meta-analysis involving 73 studies found reduced levels of vitamin D, vitamin C, magnesium, and selenium in diabetic patients with DFU compared to those without DFU [7]. Despite these findings, research investigating the association between DFU and different micronutrients remains inconclusive.

Vitamin B12 is an important micronutrient involved in various metabolic and biochemical processes. Vitamin B12 works as an intracellular antioxidant. One of the potential antioxidant properties of vitamin B12 is its ability to reduce the oxidative stress caused by advanced glycation end products (AGEs). AGEs have a significant role in the development of diabetic peripheral neuropathy (DPN) [8,9]. DPN is the most important risk factor for DFU [10]. However, the association between vitamin B12 level, DPN, and DFU is unclear and data are conflicting.

Only a few studies have investigated the relationship between vitamin B12 levels and the presence of DFU. One study, conducted in Jazan, Saudi Arabia, involving 323 adults, found a significant association between vitamin B12 deficiency and DFU [11]. Another study, conducted in Spain, found similar levels of vitamin B12 in both DM and DFU patients [12]. Additionally, a study in Australia showed the average B12 level to be within the normal range, with only 20.5% of the participants having low vitamin B12 [13]. The assessment of micronutrient status is an important but often overlooked aspect of the multidisciplinary care provided for patients with DFU, and more research is needed to establish the nutritional needs of these patients.

## Materials and methods

Study design and setting

This retrospective case-control study was conducted among Saudi adults in Buraydah who were at least 18 years old and had type 2 diabetes mellitus (T2DM). The study was conducted at the Diabetes and Endocrine Center in Buraydah, the capital city of Al-Qassim province in Saudi Arabia.

Sample size

The total sample size was 220. This size was estimated using the OpenEpi website for an unmatched case-control study with a two-sided confidence interval (CI) of 90%, a power of 80%, and a case-control ratio of 1:1. The hypothetical proportion of controls with exposure was 40% and the odds ratio was 2.

Sampling technique and inclusion and exclusion criteria

The study targeted patients with T2DM who were diagnosed with DFU and were registered at the Diabetes and Endocrine Center in Buraidah from January 2018 to August 2023. The definition of DFU included any full or partial-thickness wound below the ankle. The control group consisted of patients with T2DM registered at the Diabetes and Endocrine Center who were unaffected by DFU at the time of diagnosis of the affected cases. Any patient who was less than 18 years old was excluded.

Data collection methods

Data were collected in August 2023 from the medical records of patients who met the criteria. The data were then entered into an Excel sheet. The data included vitamin B12 levels, with a value of less than 187 pg/mL used as a cut-off point for vitamin B12 deficiency. Other data included age, sex, duration of diabetes, foot trauma, neuropathy, vasculopathy, physical activity, body mass index (BMI), HbA1c, dry foot skin, Charcot joint, and foot fissures. Any missed data were labeled as unknown.

Data sources and measurement

All data were obtained from the electronic records of the participants. The vitamin B12 level was categorized according to the center’s laboratory into the following three categories: low (less than 187 pg/mL), normal (188-883 pg/mL), and high (more than 883 pg/mL). The HbA1c was recorded as a percentage. The age, sex, and BMI were recorded from the electronic files of the participants. The diagnosis of foot ulcer, the duration of diabetes, the presence of foot trauma, neuropathy, vasculopathy, Charcot joint, and foot fissures were recorded from the clinical notes written by the physicians in diabetes and podiatric screening and follow-up clinics.

Statistical analysis

Both descriptive and inferential statistical analyses of the data were performed. Simple descriptive statistics of the sociodemographic characteristics and other clinical categorical variables were calculated and tabulated in the form of frequencies and percentages. For continuous variables, means and standard deviations (SDs) were reported as measures of central tendency and dispersion, respectively.

This involved both univariate and multivariate analyses. For the univariate analysis, the comparison of variables of interest between the controls and the cases involved an independent samples t-test and Fisher’s exact test for continuous and categorical variables, respectively. For the multivariate analysis, a binary logistic regression model was created to identify significant predictors of ulcer development in DM patients. The predictors included in the model were sociodemographic characteristics with a p-value <0.1 in the univariate analysis (age, gender), clinical characteristics (duration of diabetes, HbA1c, and vitamin B12 levels), as well as neuropathy. Other complications such as vasculopathy, dry foot, and fissures were not included due to the limited number of participants with data available for all these variables. The results of the model were presented as adjusted odds ratios (AORs) and p-values.

Significance was established at a p-value of 0.05, indicating a 95% confidence interval. All statistical calculations were performed using SPSS version 27.0.1 (IBM Corp., Armonk, NY, USA).

## Results

Sociodemographic and clinical characteristics of participants

The study included 221 participants, of whom 114 (51.6%) were controls (individuals with diabetes but without ulcers) and 107 (48.4%) were cases (individuals with diabetes and ulcers). The average age was 56.2 years (SD = 13.0), with a majority in the 51-60-year age group (29.9%), followed by the 61-70-year (24.0%) and 41-50-year (22.2%) age groups. The gender distribution was nearly equal, with 115 females and 106 males. Vitamin B12 levels varied: 175 participants (79.2%) had levels within the range of 187-883 pg/mL, 12 (5.4%) had levels below 187 pg/mL, and 34 (15.4%) had levels above 883 pg/mL. The average HbA1c was 8.1 (SD = 2.2), and the average BMI was 31.4 kg/m^2^ (SD = 7.6). More than half of the participants (58.4%) had been diagnosed with diabetes for five years or more. In terms of physical activity status, 50 (22.6%) participants were active, 43 (19.5%) were inactive, and the activity status of 128 (58.1%) participants was unknown. Neuropathy was present in 38 (17.2%) participants. Vasculopathy affected 29 (13.1%) participants, dry foot and fissures were observed in 24 (10.9%) participants, Charcot joint in 20 (9.0%) participants, and foot trauma in 20 (9.0%) participants (Table 1).

**Table 1:** Sociodemographic and clinical characteristics of the participants (N = 221). Percentages may not total 100 due to rounding. BMI: body mass index; DM: diabetes mellitus

		N	%
Type	Control (DM but no ulcer)	114	51.6%
	Case (DM with ulcer)	107	48.4%
Age (binned)	21–30	9	4.1%
	31–40	15	6.8%
	41–50	49	22.2%
	51–60	66	29.9%
	61–70	53	24.0%
	71–80	23	10.4%
	81–90	4	1.8%
	91+	2	0.9%
Gender	Female	115	52.0%
	Male	106	48.0%
Vitamin B12 level	Within the reference range	175	79.2%
	Low	12	5.4%
	More than the reference range	34	15.4%
BMI groups	Underweight (<18.5 kg/m^2^)	1	0.5%
	Healthy weight (18.5–25 kg/m^2^)	32	14.5%
	Overweight (25.0–30 kg/m^2^)	55	24.9%
	Obese (>30 kg/m^2^)	90	40.7%
	Unknown	43	19.5%
Duration of diabetes	Less than 5 years	12	5.4%
	More than or equal 5 years	161	72.9%
	Unknown	48	21.7%
Physical activity	Active	50	22.6%
	Inactive	43	19.5%
	Unknown	128	57.9%
Neuropathy	No	57	25.8%
	Unknown	126	57.0%
	Yes	38	17.2%
Vasculopathy	No	59	26.7%
	Unknown	133	60.2%
	Yes	29	13.1%
Dry foot and fissures	No	59	26.7%
	Unknown	138	62.4%
	Yes	24	10.9%
Charcot joint	No	67	30.3%
	Unknown	134	60.6%
	Yes	20	9.0%
Foot trauma	No	75	33.9%
	Unknown	126	57.0%
	Yes	20	9.0%

Factors associated with ulcer development

**Figure 1:**
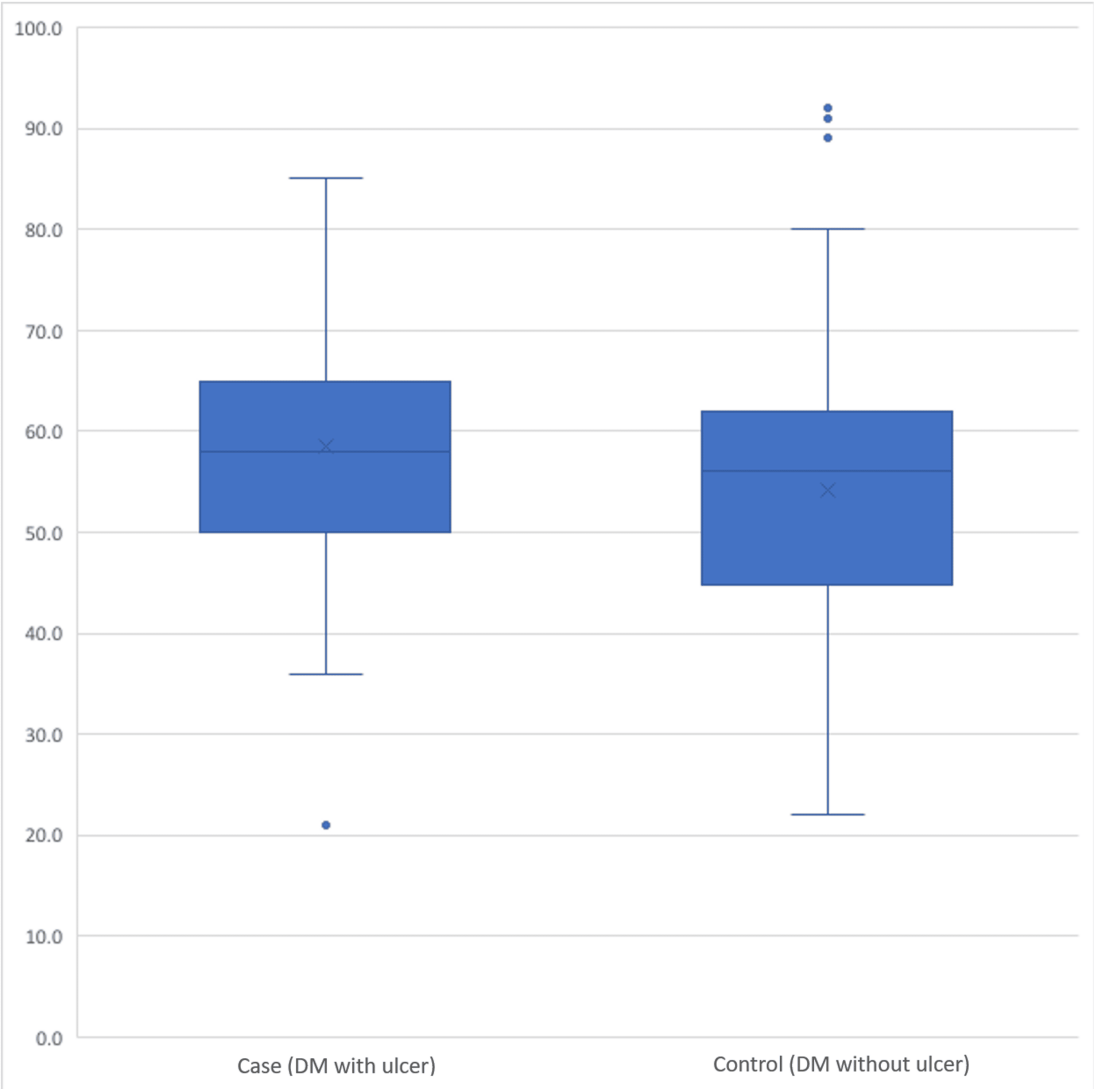
Difference in age between cases and controls (p = 0.028*). *: p < 0.05, significant. DM: diabetes mellitus

**Figure 2:**
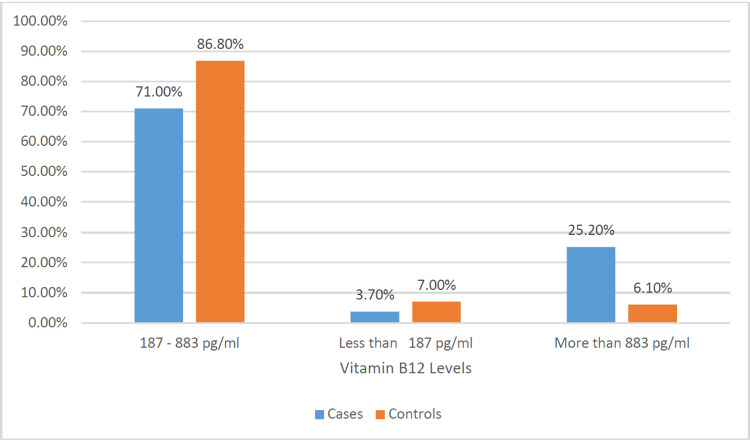
Comparison of vitamin B12 levels of cases and controls.

**Figure 3:**
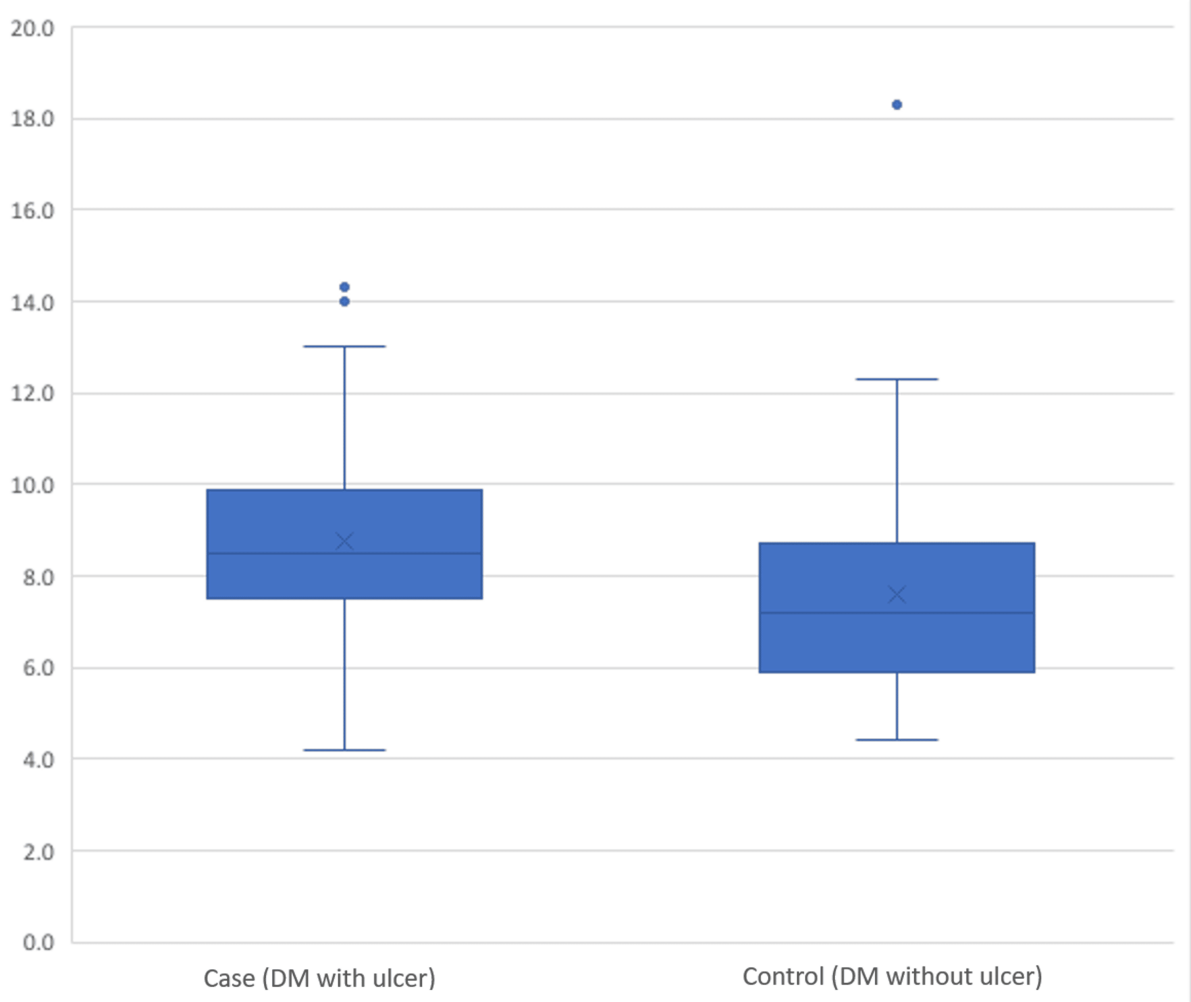
Difference in glycated hemoglobin levels between cases and controls (p = 0.014*). *: p < 0.05, significant. DM: diabetes mellitus

**Figure 4:**
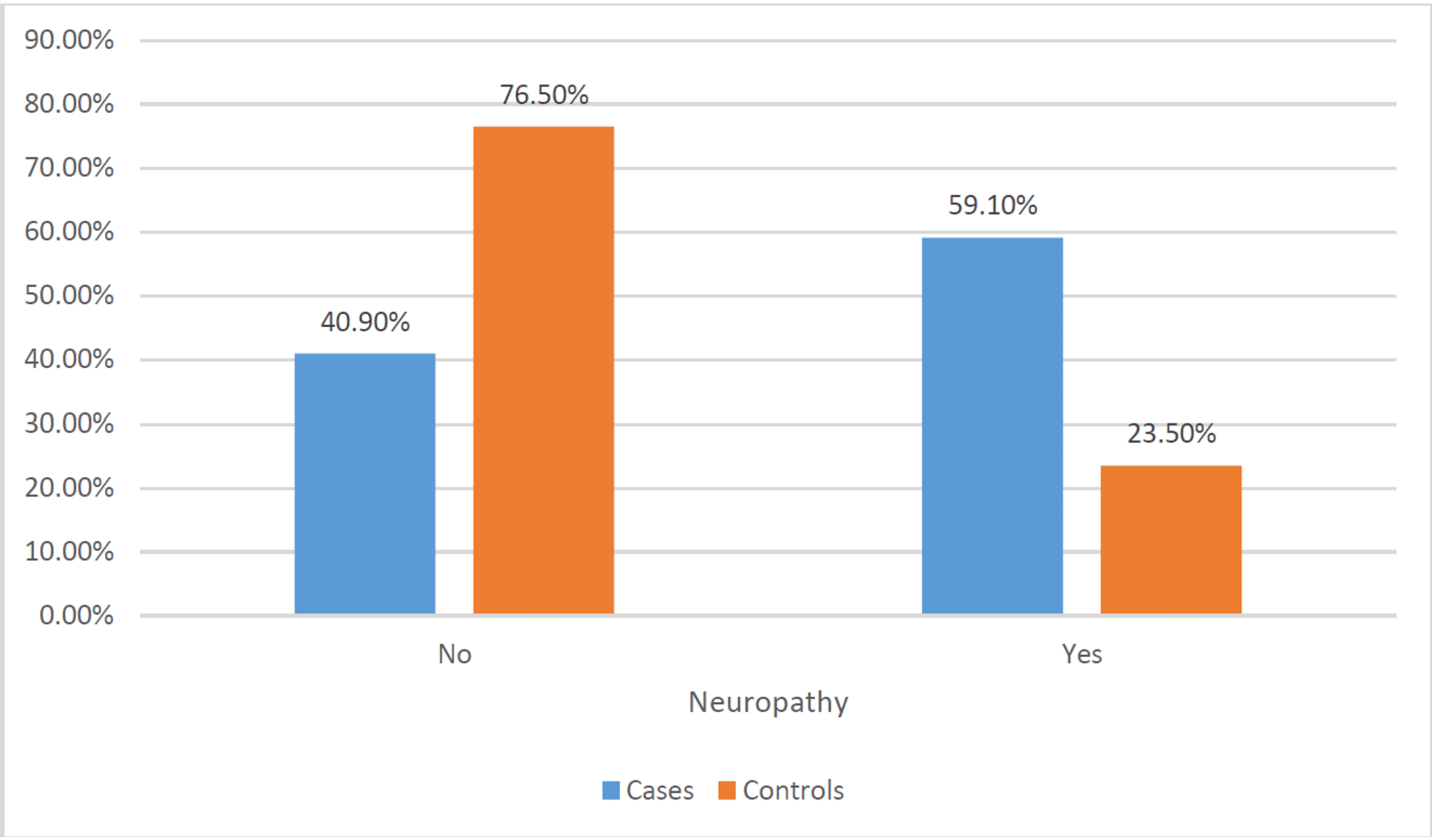
Comparison of prevalence of neuropathy in cases and controls.

A comparison between individuals with DFUs (cases) and those without ulcers (controls) revealed significant differences across various factors. The average age of cases (58.5 years, SD = 11.3) was notably higher than that of controls (54.1 years, SD = 14.1). This difference showed a significant association with ulcer development (p = 0.011) (Figure 1). While the distribution of gender did not display a significant difference, B12 levels did exhibit significance. Specifically, 25.2% of cases had levels above 883 pg/mL compared to 86.8% of controls with levels within the range of 187-883 pg/mL (p < 0.001) (Figure 2). HbA1c levels were significantly higher in cases (8.7, SD = 2.0) compared to controls (7.6, SD = 2.2) (p < 0.001) (Figure 3). Regarding physical activity, cases showed a significantly higher percentage of inactivity (62.1%) compared to controls (39.1%) (p = 0.046). Neuropathy exhibited a significant association with ulcer development, with 59.1% of cases having neuropathy compared to 23.5% in controls (p < 0.001) (Figure 4). Furthermore, complications such as dry foot and fissures (60.0% vs. 6.3%), Charcot joint (36.8% vs. 12.2%), and foot trauma (40.9% vs. 3.9%) were significantly more prevalent in cases compared to controls (p < 0.001 for all). However, vasculopathy did not exhibit a significant difference between the two groups (p = 0.492). These findings emphasize the significant associations of age, vitamin B12 levels, HbA1c, duration of diabetes, neuropathy, and specific foot complications with the development of DFUs (Table 2).

**Table 2:** Comparison of cases and controls showing factors associated with ulcer development. ^t^: Independent samples t-test; ^F^: Fisher’s exact test *: p < 0.05, significant. Percentages may not total 100 due to rounding. BMI: body mass index; DM: diabetes mellitus; SD: standard deviation

		Type								
		Control (DM but no ulcer)				Case (DM with ulcer)				P-value^t/F^
		M	SD	N	Column %	M	SD	N	Column %	
Age		54.1	14.1			58.5	11.3			0.011*
Gender	Female			66	57.9%			49	45.8%	0.081
	Male			48	42.1%			58	54.2%	
Vitamin B12 level	Within the reference range			99	86.8%			76	71.0%	<0.001*
	Low			8	7.0%			4	3.7%	
	More than the reference range			7	6.1%			27	25.2%	
Glycated hemoglobin		7.6	2.2			8.7	2.0			<0.001*
BMI		32.1	7.1			30.3	8.3			0.135
Duration of diabetes	Less than 5 years			8	8.2%			4	5.3%	0.556
	More than or equal 5 years			90	91.8%			71	94.7%	
Physical activity (unknown status excluded)	Active			39	60.9%			11	37.9%	0.046*
	Inactive			25	39.1%			18	62.1%	
Neuropathy (unknown status excluded)	No			39	76.5%			18	40.9%	<0.001*
	Yes			12	23.5%			26	59.1%	
Vasculopathy (unknown status excluded)	No			36	70.6%			23	62.2%	0.492
	Yes			15	29.4%			14	37.8%	
Dry foot and fissures (unknown status excluded)	No			45	93.8%			14	40.0%	<0.001*
	Yes			3	6.3%			21	60.0%	
Charcot joint (unknown status excluded)	No			43	87.8%			24	63.2%	0.010*
	Yes			6	12.2%			14	36.8%	
Foot trauma (unknown status excluded)	No			49	96.1%			26	59.1%	<0.001*
	Yes			2	3.9%			18	40.9%	

Predictors of ulcer development

The binary logistic regression model was designed to predict the development of ulcers in individuals with diabetes based on various predictors. Among the factors analyzed, age showed an AOR of 0.972 (p = 0.190), suggesting a non-significant association with ulcer development. The male gender had an AOR of 1.030 (p = 0.952), indicating no significant effect. Similarly, HbA1c levels showed an AOR of 1.137 (p = 0.378), suggesting a non-significant association. However, vitamin B12 levels above 883 pg/mL demonstrated a significant positive association with ulcer development, with an AOR of 3.874 (p = 0.050). A diabetes duration of more than five years had an AOR of 0.544 (p = 0.504), which was not statistically significant. Notably, the presence of neuropathy emerged as a highly significant predictor of ulcer development, with an AOR of 4.317 (p = 0.004). This analysis highlights the critical role of neuropathy and elevated vitamin B12 levels in predicting the occurrence of DFUs, emphasizing the importance of monitoring and early intervention in individuals exhibiting these risk factors (Table 3).

**Table 3:** Binary logistic regression model for prediction of ulcer (N = 88). *: p < 0.05, significant.

Predictors		Adjusted odds ratio	P-value
Age		0.972	0.190
Male gender		1.030	0.952
Glycated hemoglobin		1.137	0.378
Vitamin B12 level (pg/mL)	Within the reference range	Ref	0.137
	Low	0.817	0.857
	More than the reference range	3.874	0.050*
Diabetes duration of more than 5 years		0.544	0.504
Neuropathy present		4.317	0.004*

## Discussion

The association between vitamin B12 deficiency and DFU is not well established, with only a limited number of studies focusing on this subject. Our study aims to assess the relationship between vitamin B12 deficiency and the development of DFUs, along with other factors. This will hopefully aid in guiding future preventive and screening measures, as well as the management of DFUs.

Diabetes can lead to widespread and severe illnesses below the knee. A DFU carries a lifetime risk of between 19% and 34%. It is estimated that between 9.1 million and 26.1 million people with diabetes worldwide experience foot ulcers each year [14]. Nearly 50% of patients with DM experience a diabetic foot (DF) [15]. In Saudi Arabia, patients aged 55 and older who have experienced long-term complications linked to severe or uncontrolled DM account for more than 44% of patients [16,17]. The results of our study indicate no significant association between vitamin B12 deficiency and DFUs. However, it does emphasize the role of peripheral neuropathy as a predictive factor for ulcer development and supports the observed associations that align with the broader diabetes literature regarding other factors contributing to the development of DFU.

Contrary to the expected association, the prevalence of vitamin B12 deficiency, defined as levels below 187 pg/mL, was unexpectedly low at 5.4%, with the majority of participants falling within the reference range of 187-883 pg/mL. This low prevalence could be attributed to several factors, including increased dietary consumption of vitamin B12-rich foods among the studied population and potentially increased supplementation with vitamin B12 in diabetic patients [18]. However, the surprisingly low percentage of participants with vitamin B12 deficiency, particularly in contrast to the high proportion diagnosed with peripheral neuropathy, challenges the previously observed association between these two factors [19]. Interestingly, 25% of the participants demonstrated abnormally high serum B12 levels, exceeding 883 pg/mL. According to Zulfiqar (2019), this finding constitutes a biological abnormality that has been linked to various conditions such as solid neoplasia, hematological disorders, liver disease, and renal failure [20]. Notably, renal failure is a common complication of diabetes [21].

The study further underscores the role of neuropathy in predicting the occurrence of DFUs [22], a risk factor extensively studied for DFU. The effects of peripheral neuropathy include impaired balance caused by a loss of proprioception, decreased sweating, and xerosis that can crack and fissure, which increases the risk of developing DFUs. When compared to diabetics without neuropathy, those with moderate-to-severe sensory loss have a seven-fold increased risk of getting their first foot ulcer [23].

The study also highlights several other key factors associated with the development of DFUs. Age emerges as a significant contributor, with older individuals being more prone to ulcer development. This is possibly due to older individuals having a longer duration of diabetes along with decreased levels of activity. Elevated HbA1c, which is strongly associated with multiple complications of diabetes [24], longer diabetes duration, and specific foot complications, further highlights the complex interplay of ulcer development in individuals with diabetes.

The results of this study have several significant implications for both clinical practice and public health. It is recommended that clinicians monitor vitamin B12 levels, particularly in patients who are at risk of developing vitamin B12 deficiency [25]. Regular screening for neuropathy and specific foot complications is essential for timely intervention [26]. The implementation of targeted strategies to address identified risk factors, such as optimizing B12 levels and managing neuropathy, could potentially help in preventing or mitigating the occurrence of DFUs. Public health initiatives could be enhanced by increasing awareness about the importance of micronutrient status [27,28], thereby emphasizing the need for a holistic approach to diabetes management [29].

Future research in this domain could extend the current study by incorporating longitudinal designs to establish causality and gain a deeper understanding of the dynamic relationship between vitamin B12 levels and DFUs over time. An investigation into the effects of dietary patterns and lifestyle factors on B12 status and ulcer development could offer a more holistic understanding of these associations. Further studies are required to evaluate the signs and symptoms of vitamin B12 deficiency, particularly in patients with normal vitamin B12 levels, as functional cobalamin deficiency can occur at any serum level [30]. Additionally, broadening the study to include diverse geographic regions and larger sample sizes would enhance the applicability of the findings. The exploration of potential interventions to address vitamin B12 deficiencies and neuropathy in diabetic populations could further aid in the formulation of targeted preventive strategies.

While this study provides valuable insights into the association between vitamin B12 deficiency and DFUs, several limitations warrant consideration. First, the retrospective design of the research implies that causation cannot be definitively established, thereby limiting the ability to infer a direct relationship between vitamin B12 levels and the development of ulcers. Second, the study’s focus on a single geographic region, Qassim, may limit the applicability of the findings to broader populations. Although the sample size of 221 participants is informative, it may not fully capture the range of factors influencing DFUs. Lastly, the lack of longitudinal data restricts the examination of how fluctuations in vitamin B12 levels over time might affect ulcer development.

## Conclusions

The observed associations with age, HbA1c, duration of diabetes, neuropathy, and specific foot complications highlight the multifactorial nature of ulcer development. The logistic regression model identifies neuropathy as a significant predictor of ulcer occurrence. Our study concluded that most participants with DFUs have vitamin B12 levels within the normal range. This study offers practical implications for clinicians and suggests directions for future research aimed at refining preventive measures in the field of diabetic foot care.
